# Combating trafficking in persons: a call to action for global health professionals

**DOI:** 10.9745/GHSP-D-13-00142

**Published:** 2014-07-08

**Authors:** Luis CdeBaca, Jane Nady Sigmon

**Affiliations:** a U.S. Department of State, Washington, DC, USA

## Abstract

Health care professionals can help identify victims of human trafficking, who commonly come into contact with providers during captivity. Providers can also help restore the physical and mental health of trafficking survivors. Training should focus on recognizing trafficking signs, interviewing techniques, and recommended responses when a victim is identified.

Trafficking in persons—also known as modern slavery—is a crime that undermines the most basic rights of an individual. Despite more than a decade of international and domestic laws against it, human trafficking affects every country in the world, including the United States. As global awareness of human trafficking has grown, efforts to combat it have also increased. An Internet search using the term “human trafficking” yielded more than 32 million results in seconds. Awareness of the impact of this crime on victims and of its various manifestations has also evolved. Victims have been found in, and freed from, slave-like conditions in nearly every industry: agriculture, manufacturing, construction, hospitality, health care, janitorial services, mining, fishing, domestic service, as well as commercial sex. The scale of the problem has prompted professionals and advocates to embrace the notion that all sectors must be equipped with the knowledge and relevant skills to contribute to ending modern slavery.

Social science experts estimate there are 20.9 million^1^ to 29.8 million^2^ people around the world living in servitude. A report from the International Labour Organization (ILO) provided additional information about the victims of human trafficking^1^:

An estimated 21 million to 30 million people worldwide are living in servitude.

- Of the total number of 20.9 million victims, 18.7 million (90%) are exploited in the private economy by individuals or enterprises; the remaining 2.2 million are in state-imposed forms of forced labor. Of those exploited in the private economy, 4.5 million (22% of the total number) are victims of forced sexual exploitation, and 14.2 million (68% of the total number) are victims of forced labor exploitation.
- Women and girls comprise the majority of victims—11.4 million (55%)—representing nearly all the victims of forced sexual exploitation and approximately 40% of the victims of forced labor exploitation.
- Children represent approximately one-quarter (26%) of the victims of human trafficking.
- Victims spend, on average, approximately 18 months in forced labor.

The majority of victims of human trafficking are women and girls.

Each victim, regardless of gender, age, sexual orientation, ethnicity, or country of origin, has his or her own personal story. For example:

*Vannak Anan Prum was lured from Cambodia to Thailand by the promise of a lucrative job, but instead he was deceived by a labor broker. He was forced to work on a Thai fishing vessel from 2005 to 2009 in slave-like conditions, never receiving a salary. Mistreated, starved, and tortured … Mr. Prum escaped with another fisherman by jumping off the boat and swimming four kilometers to shore when the boat anchored off Malaysian Borneo. According to his account, upon attempting unsuccessfully to obtain help returning to Cambodia, he was sold by corrupt officials to a palm oil plantation. After several months of forced labor on the plantation, an altercation with another worker landed him in detention. While [there], he was able to establish contact with Malaysian and Cambodian human rights NGOs, which collaborated to have Mr. Prum repatriated to Cambodia, though not until he had spent several additional months in detention.*^3^

Since regaining his freedom, Vannak Prum—like many survivors of abuse—has become an advocate, committed to preventing others from suffering a similar fate. He has worked to raise awareness of labor trafficking in the Thai fishing industry through a series of intricate drawings of his experience, and he was honored by U.S. Secretary of State Hillary Clinton as a “2012 Trafficking in Persons Report Hero” for his tireless efforts to end modern slavery. Mr. Prum's efforts are an example of survivor activism that, along with the advocacy of governments, nongovernmental organizations (NGOs), and multilateral organizations, has contributed to the formation of a global movement.

This article is a call to action for clinicians and public health professionals to engage in the response to human trafficking globally. It provides an overview of the prevalence of the crime, terminology, its common manifestations and health consequences, advancements in addressing it, and suggestions for how global health professionals can contribute to ending modern slavery.

## WHAT IS HUMAN TRAFFICKING?

“Trafficking in persons” and “human trafficking” are used as umbrella terms for all acts involved in recruiting, harboring, transporting, providing, or obtaining a person for compelled service or commercial sex acts through the use of force, fraud, or coercion.^4^ Since 2000, the United States *Trafficking Victims Protection Act of 2000* (TVPA)^5^ and the United Nations *Protocol to Prevent, Suppress and Punish Trafficking in Persons, Especially Women and Children* (Palermo Protocol)^6^ have described this compelled service using several overlapping concepts, including involuntary servitude, slavery, practices similar to slavery, debt bondage, and forced labor. Under both the TVPA and the Palermo Protocol, if commercial sex involves a minor, then force, fraud, or coercion does not need to be present. The TVPA and the Palermo Protocol also embrace a victim-centered approach to the crime of human trafficking and adopt the “3P Paradigm” as the international framework for responding to human trafficking:

- Prosecution of traffickers
- Protection and assistance for victims
- Prevention of trafficking from occurring

In recent years, a fourth “P”—Partnerships—has been recognized by many as the critical ingredient to establishing comprehensive responses to human trafficking.

In the past decade, there has been greater understanding of the trafficking phenomenon and manifestations of the crime. For example, exploitation and compelled service and the coercive and deceptive practices used by traffickers are at the heart of the many forms of modern slavery. Individuals may be victims of trafficking regardless of whether they once consented to work for the trafficker, participated in a crime as a direct result of being trafficked, were transported into the exploitative situation, or were simply born into a state of servitude. And although the term “trafficking” suggests movement, the crime does not require movement. Many people are enslaved in their own communities or in cities within their country of origin.

## GOVERNMENT RESPONSES TO HUMAN TRAFFICKING

The United States emerged as a world leader on this issue in 2000, when comprehensive anti-trafficking legislation, the TVPA, was passed, which authorized establishment of the Office to Monitor and Combat Trafficking in Persons in the U.S. Department of State (TIP Office). The TIP Office compiles an annual report to Congress assessing governments' efforts to implement anti-trafficking laws and policies. The 2014 *Trafficking in Persons Report* (TIP Report) contains rankings and individual country narratives for 188 countries, including the United States.^7^

Since 2000, a remarkable international consensus has developed regarding the need to address human trafficking. Most governments have taken concrete steps to create the legal framework to address this crime: 159 countries have become parties to the Palermo Protocol,^6^ and 133 countries prohibit all forms of trafficking.^8^ Even with this progress, human trafficking remains a hidden crime, and the identification of victims of human trafficking continues to be a fundamental challenge. As a consequence, millions of victims suffer in grossly exploitative situations every day, and only a small number of the victims held in compelled service have been given the assistance and services they need. Figures gathered for the 2014 TIP Report^7^ show an enormous gap between the estimated number of victims globally (as many as 29.8 million) and the estimated number of victims actually identified globally (only approximately 44,800). In addition, most traffickers are not apprehended and punished. Fewer than 9,500 prosecutions were reported globally, resulting in approximately 5,800 convictions.

Most countries prohibit human trafficking, but it remains a hidden crime.

Several factors have contributed to under-identification of victims—including those who are still in the trafficking situation and those who have been freed. Traffickers maintain control over victims through isolation, debt bondage, deception, violence, and coercion, including threats against them or their families, false promises of future pay for work already done, and threats of arrest or deportation. Unaware of their rights and of the existence of protections for trafficking victims, many victims do not come forward to identify themselves and are treated by authorities as criminals, illegal migrants, people in prostitution, or juvenile delinquents.^9^^,^^10^

After 2000, initial programmatic and enforcement efforts to combat human trafficking focused primarily on sex trafficking of women and girls across national borders. Programs to assist female survivors increased in number, and they developed comprehensive services, including shelter, health care, counseling, legal advocacy, and assistance with repatriation and community reintegration. Less attention was given to trafficking of men and boys. Research shows that male trafficking victims are often not recognized due to commonly held beliefs or assumptions that trafficking is about women who are held in sexual servitude, whereas exploited men are seen as irregular migrants who should be deported without investigating their circumstances.^11^ Interviews with male victims have provided examples of how numerous professionals (doctors, border guards, police, and prison officials) have failed to identify male victims, even when the men described what had happened to them.^12^^,^^13^

## HEALTH CONSEQUENCES OF HUMAN TRAFFICKING

The physical and mental health consequences of human trafficking have become clearer through systematic research and through the work of NGOs who address the care and assistance needs of trafficking survivors. One study found that more than half of female survivors of sex trafficking seeking services (59%) reported sexual or physical violence prior to the trafficking experience; nearly all (95%) reported physical or sexual violence during the trafficking situation.^14^ Most of the survivors (57%) reported physical injuries, and the vast majority (76%) reported they were never able to do as they wished or go where they wanted. In the first 2 weeks of their post-trafficking care, the majority of respondents reported physical symptoms including: headaches (82%), fatigue (81%), dizzy spells (70%), back pain (69%), memory difficulty (62%), pelvic pain (59%), and gynecological infections (58%). Most (63%) reported more than 10 concurrent physical health problems. Additional research on female survivors of sex trafficking showed comorbidity for 3 mental health outcomes—anxiety, depression, and post-traumatic stress disorder (PTSD)—and the severity of symptoms was associated with the length of time spent in the trafficking situation.^15^ Risk of HIV infection is also an issue. Both young age when first becoming a victim of sex trafficking and length of time in the brothel were found to heighten the risk of becoming infected with HIV.^16^ A systematic review found HIV prevalence among trafficked women ranging from 22.7% to 45.8% and documented the high prevalence of physical symptoms when trafficked women come into care.^17^ The presence of multiple traumas experienced by many sex trafficking victims has implications for interventions, treatment, and planning for the safe return and reintegration of survivors.

Systematic research on the health impact of trafficking on male victims is not yet available, but recent research indicates that many male victims of forced labor experience violence and need access to physical and mental health services. In a recent case series report of 35 adult victims of forced labor in the United Kingdom who received services post-trafficking (27 of whom were men), 40% experienced physical violence (for example, being kicked, hit, hurt with a gun or knife, or intentionally burned) while in the trafficking situation, and 81% reported one or more symptoms of poor physical health.^18^ In other research, interviews with male survivors of forced labor show that many experience harsh and traumatizing treatment from their exploiters: physical abuse and assaults, threats of violence, loss of control over basic life functions, and loss of freedom of movement.^12^^,^^19^ Survivors reported being forced to live in cramped, locked settings, with poor diet and sanitation, and some are exposed to the elements. Anecdotal reports from NGOs and law enforcement in the United States also show that forced labor cases may include exploitation of drug or alcohol dependency, mental illness or disability, and sexual assault or threats of sexual assault as methods of control. Commercial sexual exploitation of boys and men has received less attention, but cases have been reported in Kenya, Southeast Asia, Spain, and the United States, and it is widely suspected that such cases are significantly underreported.^20^

## HEALTH RESPONSES AND RESOURCES

Human trafficking is increasingly recognized as a global public health problem, and guidance for health care providers has emerged in the literature^21^^,^^22^ as well as calls for the development and implementation of new education and training programs for health care professionals.^23^ In addition, there is evidence that victims of human trafficking commonly come into contact with a health care provider during their captivity and exploitation, but too often these encounters represent missed opportunities for victim identification and assistance.

Through interviews with survivors, 2 studies in the United States have explored the experiences of sex trafficking and/or forced labor victims who received medical or dental care during the time they were being exploited. Of the 21 survivors interviewed in one study, 28% had come into contact with health care providers.^24^ In the other study, half of the 12 survivors had received health care.^25^ Victims of involuntary domestic servitude were taken for treatment for respiratory or systemic illnesses or bodily injury that prevented them from performing household duties. Sex trafficking victims saw health care professionals for sexually transmitted infections and abortions. Victims were seen in a variety of settings, from small clinics and private offices to large public medical facilities. These studies show, and experienced practitioners observe,^26^ that victims are not likely to be taken for health care until the condition becomes serious. They also note several obstacles to a victim's disclosure of the trafficking situation, including fear for themselves or their families, shame, a language barrier, concern that they would be ridiculed or not believed, and the limited interaction between the victim and health care staff. This limited interaction can often be attributed to the behavior of traffickers who accompany and speak on behalf of the victim or seek to monitor or control the victim's communication during the health care visit.

To ensure that health care services are not a missed opportunity for victim identification, training must go beyond raising awareness of the plight of victims and recognizing the signs of human trafficking. It should also cover guidance on interacting with potential victims, interviewing techniques, recommended responses, and resources.^27^ A literature review^28^ of recommended strategies for U.S. human service agencies (such as health care, child welfare, social service, juvenile justice, domestic violence, and victim advocacy) that engaged in identifying victims of sex trafficking found consistency in the literature regarding human trafficking indicators, for example:

- Signs the person is being controlled by someone who is accompanying him or her
- Signs the person does not have freedom to exit a job or move
- Signs of physical abuse
- Signs indicating a person is fearful or depressed

There was less agreement in the literature, however, about what a human service provider should do in response to a suspected case, including how providers should interact with potential victims, how they should screen for sex trafficking safely and sensitively in the context of a single encounter, and what their immediate response should be once a sex trafficking victim is identified. Acknowledging that these are topics for further practice development and research, studies agree that human service providers should have more training on victim identification and resources for trafficking victims before undertaking screening strategies.

A publication designed for an international audience of health care providers, *Caring for Trafficked Persons: Guidance for Health Providers*,^29^ provides more detailed guidance to help all types and levels of health care providers meet the challenges of diagnosing and treating trafficked persons. It discusses the health problems associated with sex trafficking and labor trafficking, the risks and safety issues when encountering a suspected trafficking situation, and safe and appropriate approaches to providing health care for trafficked persons. This guide was a collaborative effort between the International Organization on Migration (IOM) and the Gender Violence and Health Centre of the London School for Hygiene and Tropical Medicine and was supported by the United Nations Global Initiative to Fight Trafficking in Persons (UN.GIFT). It has been translated into Arabic, Chinese, and Spanish. Building on this effort, the U.S. State Department's TIP Office supported the development of the companion training facilitator's guide, *Caring for Trafficked Persons: Guidance for Health Providers, Facilitator's Guide*,^30^ to promote wider training of health care professionals globally.

Increasingly local and national governments are encouraging the involvement of health care providers in victim identification and are providing them with information and guidance on the signs of human trafficking, recommended screening procedures, and how to obtain/access resources. For instance, the Belgian government has a cooperation project with hospitals to improve detection of trafficking victims who may be seeking medical treatment; preliminary findings of the project indicate that victims are more willing to talk to medical staff than to police.^20^ Examples of government dissemination of information and resources include:

- U.S. Department of Health and Human Services, Rescue and Restore campaign with online toolkits for health professionals (http://www.acf.hhs.gov/programs/orr/resource/rescue-restore-campaign-tool-kits)
- UK National Health Service publication, *Identifying and Supporting Victims of Human Trafficking: Guidance for Health Staff* (www.gov.uk/government/publications/identifying-and-supporting-victims-of-human-trafficking-guidance-for-health-staff)

CAST (Coalition to Abolish Slavery & Trafficking), a Los Angeles-based NGO that was presented the Presidential Award for Extraordinary Efforts to Combat Trafficking in Persons by U.S. Secretary of State John Kerry in 2014, is also working to expand the knowledge base of health professionals working on human trafficking and to link them with others doing similar work. CAST supports the exchange of trafficking and health-related information across an interdisciplinary network of health professionals (physicians, nurses, dentists, psychologists, counselors, public health workers, health educators, researchers, social workers, administrators, and other health professionals) through its website: www.castla.org/trafficking-and-health. The purpose is to share best practices, expand evidence-based practices, and promote improved systems of care for victims of human trafficking. Interested professionals can also join an affiliated Human Trafficking and Health Care listserv (at http://www.humantraffickingandhealthcare.com) that uses Google Groups to facilitate communication within this online community.

## THE ROLE OF GLOBAL HEALTH PROFESSIONALS

Restoring the physical and mental health of trafficking survivors is a critical part of protection and assistance services, and the role of global health professionals in meeting this challenge is evolving rapidly. Researchers and clinicians have called for more specialized education and training for health care professionals, the development of new protocols for the identification of trafficking victims in health care settings, culturally sensitive and safe procedures for responding when a victim is identified, and the provision of comprehensive care post-trafficking.

While modern slavery is unique in its manifestations and impact on victims, global health professionals are encouraged to build on lessons learned from decades of experience shaping the public health response to other forms of abuse, such as domestic violence, in order to improve and expand upon current practices. In addition, global health professionals are uniquely positioned to conduct research on the epidemiology of human trafficking, as well as to evaluate the effectiveness of various treatment approaches and direct services provided to victims of trafficking. Depending upon their specialty and position, global health professionals can provide leadership and can contribute in numerous ways to improve the response to human trafficking, only a few of which are listed below:

- Become informed about the contexts in which human trafficking is found today and be able to identify a person who may be a victim of human trafficking.
- Develop and teach human trafficking courses in education and training programs for health professionals who serve in a wide range of health care settings and who may come into contact with victims of human trafficking or be called on to support services for victims. Such training is needed for the full range of health professionals, including physicians, nurses, physician assistants, dentists, psychologists, social workers, drug abuse counselors, health administrators, and others.
- Build on the existing body of human trafficking-related research and evaluation by conducting studies that examine key health issues resulting from human trafficking and that explore effective means of health care delivery.
- Become informed about local laws and government policies and procedures related to human trafficking, and identify ways to improve health-related responses and the delivery of comprehensive, coordinated services for survivors.
- Establish linkages with interagency partners that have responsibility for policies and procedures related to human trafficking, and participate in interagency task forces and other efforts aimed at developing coordinated, interdisciplinary anti-trafficking protocols.
- Work to develop trauma-informed policies and procedures in health care delivery settings that ensure recognition of the signs of human trafficking, the establishment of protocols to follow for suspected cases of human trafficking, linkages with appropriate resources, and staff training to ensure implementation.
- Work with NGOs and faith-based communities that are providing services to survivors of human trafficking to help expand and improve services to address the physical and mental health needs of survivors.
- Join or create an online network of health professionals to share information on challenges and advances in health care responses to human trafficking.

## CONCLUSION

Vannak Prum's journey to freedom described earlier is an inspiration, and his unique contributions to ending modern slavery reflect the resilience displayed by many survivors of horrendous abuses suffered at the hands of traffickers. His experience also highlights the challenges we face going forward. From the time he escaped the fishing boat on which he was enslaved, Mr. Prum encountered police, nurses, doctors, and jailers who did not recognize his circumstances to be those of human trafficking. Most of the people with whom he came in contact did not see him as a trafficking victim in need of help, but rather as an illegal alien, a migrant worker, or an arrestee. The tragedy of Mr. Prum's situation going unrecognized, untreated, and unserved is repeated countless times every day around the world. With in-depth training, improved protocols, and enhanced interagency coordination, health professionals can change previously missed opportunities into concrete steps toward our common goal—a world without slavery.

## Notes

**Peer Reviewed**

**First published online:** 2014 Jul 8
